# Targeting the 4-1BB costimulatory molecule through single chain antibodies promotes the human T-cell response

**DOI:** 10.1186/s11658-020-00219-8

**Published:** 2020-04-22

**Authors:** Salman Bagheri, Elmira Safaie Qamsari, Mehdi Yousefi, Farhad Riazi-Rad, Zahra Sharifzadeh

**Affiliations:** 1 Department of Immunology, Pasteur Institute of Iran, Tehran, Iran; 2 Department of Immunology, School of Medicine, Tabriz University of Medical Sciences, Tabriz, Iran; 3 Immunology Research Center, Tabriz University of Medical Sciences, Tabriz, Iran

**Keywords:** 4-1BB, Single-chain fragment antibody, T-cell therapy, Immunomodulation, T cell responses

## Abstract

**Background:**

Adoptive T-cell therapy (ACT) using autologous tumor-reactive T lymphocytes has considerable potential for cancer immunotherapy. In ACT, T cells are isolated from cancer patients and then stimulated and expanded in vitro by cytokines and costimulatory molecules. 4-1BB is an important costimulatory protein belonging to the TNF receptor superfamily. It is involved in T-cell survival, proliferation and activation. Agonistic anti-4-1BB monoclonal antibodies have been introduced as appropriate tools for ACT.

**Methods:**

Here, various single-chain fragment variable (scFv) antibodies were used to activate T cells isolated from peripheral blood via immune magnetic isolation. The T cells were stimulated with IL-2 and anti-CD-3 mAb and then treated with agonistic anti-4-1BB scFvs. The results showed the remarkable effects of anti-41BB scFvs on the functional properties of T cells, including their activation, proliferation and cytokine production. The flow cytometry analysis revealed a considerable increase in the expression of the T-cell activation marker CD69. Moreover, T-cell proliferation was evidenced in treated cells by CFSE labeling compared to the control groups.

**Result:**

Anti-4-1BB scFvs significantly increased IFN-γ and IL-2 mRNA and protein expression in T cells, but exhibited no stimulatory effect on IL-4 expression. These findings show that anti-4-1BB scFvs could evoke a Type I immune response.

**Conclusions:**

Our results demonstrate that targeting the 4-1BB molecule using agonistic scFvs could be an effective strategy for T-cell stimulation as part of an ACT approach to cancer treatment.

## Background

4-1BB (CD137; TNFRSF9) is an inducible costimulatory molecule. It and its ligand were discovered in the 1980s in activated T cells and antigen-presenting cells (APCs) [1, 2]. 4-1BB, a Type I membrane glycoprotein, is a member of the tumor necrosis factor receptor (TNFR) superfamily. It augments cellular immunity via signal transmission through protein–protein interactions that either extend survival or enhance costimulatory signals. The 4-1BB gene is localized on chromosome 1p36, close to other TNFR family members, including TNF-RII, OX40 and CD30. T-cell activation upregulates the expression of 4-1BB [3–5].

4-1BB is induced within 24 h of activation. Signaling through the T-cell receptor (TCR) or CD3 can stimulate it on T cells [4, 6, 7]. Its expression has also been found on NKT cells, monocytes, macrophages, activated B cells, dendritic cells, eosinophils, neutrophils, epithelial and hepatoma cells, CD11^+^ dendritic cells and regulatory T cells [8].

4-1BB binds to its ligand (4-1BBL or CD137L), a transmembrane molecule of the TNF family that is expressed by APCs. 4-1BBL is induced after cell activation and can be regulated by LPS, Ig or CD40 signals [4, 6, 7, 9]. In addition to T-cell costimulation through the 4-1BB receptor, 4-1BBL has the ability to enhance the activation and proliferation of APCs via reverse signaling [7, 10]. Multiple studies have shown that 4-1BB acts as a costimulatory molecule for T-cell activation. The costimulatory signal provided by 4-1BB is involved in many T-cell responses, including tumor immunity, allograft rejection and viral infection [11–13]. 4-1BB signals can costimulate T cells by activating the NF-κB, c-Jun and p38 downstream pathways independently of CD28 signals. It has been shown that 4-1BB signaling can activate the transcription of several genes with immune system involvement, such as those for T-cell expansion and those coding for interleukin-2 (IL-2) and IFN-γ [7, 14–16].

The biological effects of 4-1BB are varied and include the upregulation of anti-apoptotic signals in T cells, the prevention of activation-induced cell death (AICD), the facilitation of differentiation into effector and memory cells, and the cell cycle progression and proliferation of T cells. In addition, it has been shown that 4-1BB signaling enhances TNF-α and IL-8 production by monocytes and can ameliorate AICD of neutrophils [4, 6, 7].

Immunotherapy, chemotherapy and radiotherapy are used individually or in combination for the treatment of cancer, autoimmune diseases and other disorders. Adoptive cell therapy (ACT) is a treatment method in which T-cell populations from patients are expanded in vitro in the presence of activating molecules, and then returned to the body. This approach relies on the in vivo development of sufficient numbers of natural host T cells with anti-tumor reactivity or host T cells genetically engineered with tumor-specific T-cell receptors (TCRs). T cells that are infused back into a patient after in vitro expansion can journey to the tumor and mediate cancer regression [17–19].

ACT has multiple advantages over other forms of cancer immunotherapy. It has been proven to be a safe and successful approach for establishing sustained T-cell responses. The infusion of small numbers of specific T cells could result in T-cell expansion in vivo and give rise to long-term anti-tumor repression [17, 20]. A major hurdle to the development of ACT is the AICD of T cells and the loss of necessary molecules and specific costimulatory signaling pathways due to the in vitro culture conditions. This leads to reduced in vivo persistence after adoptive transfer [21]. It was found that CD8 tumor-infiltrating lymphocytes (TILs) upregulate costimulatory molecules of the TNF-R family, especially 4-1BB and, to a lesser extent, CD134/OX40, and lead to a loss of CD27 and CD28 expression during initial TCR stimulation [18, 22].

It has been shown that the agonistic monoclonal antibodies (mAbs) against 4-1BB could significantly inhibit AICD in T cells, increase their proliferation and survival, and enhance their cytotoxicity. Moreover, they increased the yield of CD8 T cells and enhanced effector memory properties [18, 23].

Costimulation through the 4-1BB pathway protects human melanoma tumor-infiltrating lymphocytes from AICD and significantly enhances their anti-tumor effects [23]. It has also been proven that 4-1BB^+^ TILs mediate higher anti-tumor effects in vivo, compared with 4-1BB- TILs, which reveals the important role of 4-1BB in anti-tumor immunity [24]. The application of anti-4-1BB mAbs in clinical trials alone or in combination with cytotoxic T lymphocytes (CTL) yielded promising clinical outcomes. Their effective synergism resulted in complete eradication and growth control of melanoma tumors [25]. Various therapeutic strategies have been investigated for the stimulation of 4-1BB, and their promise in cancer immunotherapy has been demonstrated [26, 27].

We previously used phage display technology to isolate four anti-4-1BB single-chain fragment variable antibodies (scFvs), called PI.12, PI.42, PII.16 and PII.29 [28]. These antibody fragments specifically bind to 4-1BB, activate T cells and increase IL-2 production. In an attempt to generate T cells with improved biological activity, we treated human T cells with anti-4-1BB scFvs and studied their activation status, proliferation rate and cytokine production. The results of this research indicate that these scFvs are promising tools to produce T cells suitable for adoptive cell therapy.

## Materials and methods

### Isolation of T cells from whole blood

**Fig. 1:**
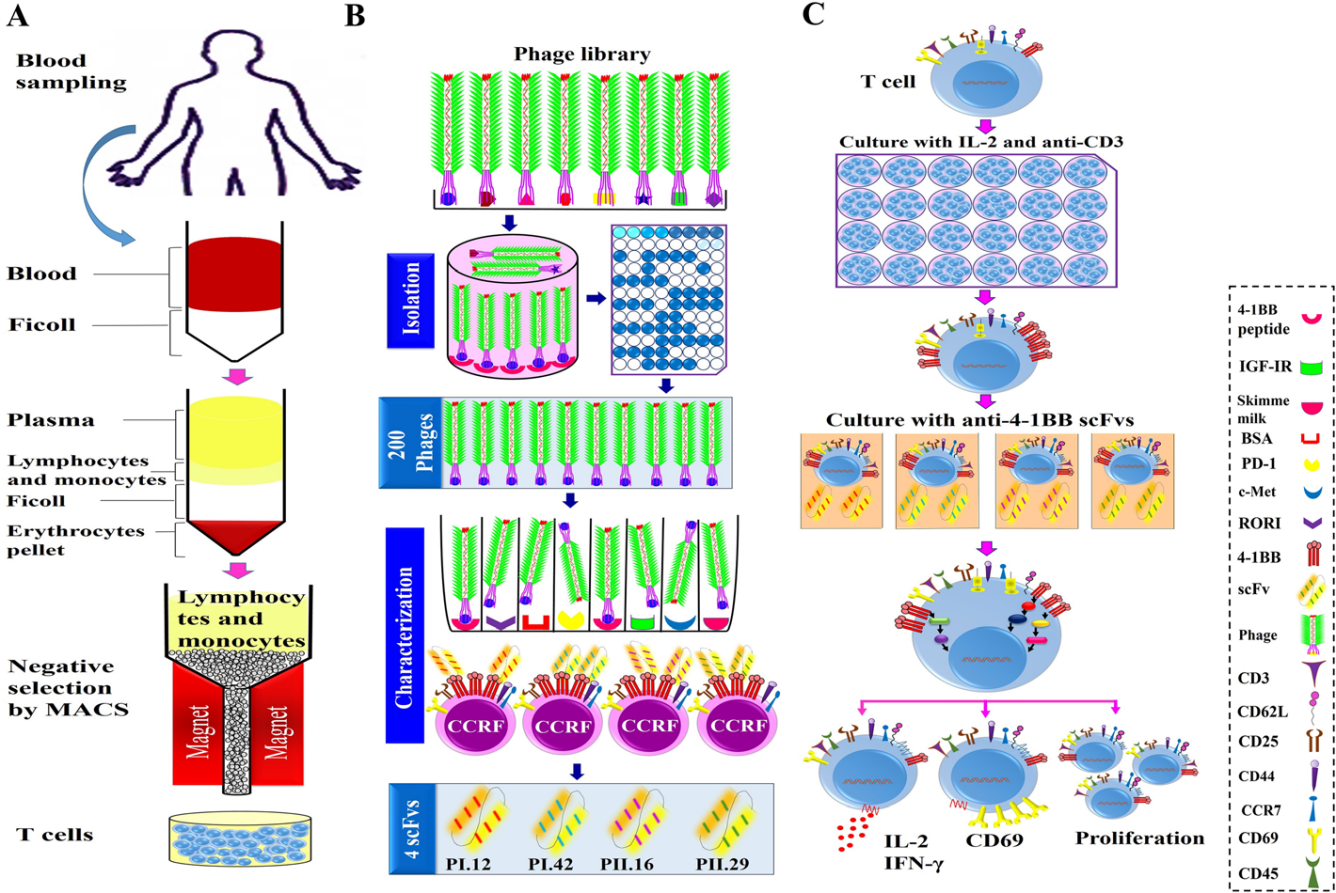
Schematics describing the isolation of human T cells, selection of anti-4-1BB single chain fragment variable antibodies (scFvs), and T-cell treatment. **a** Isolation of CD3^+^ T cells from peripheral blood via ficoll and negative selection (depletion of unwanted cells) using magnetic affinity cell sorting (MACS). **b** Isolation and characterization of four anti-4-1BB scFvs (PI.12, PI.42, PII.16 and PII.29) via phage display technology. **c** Stimulation of T cells with IL-2 and anti-CD3, treatment with anti-4-1BB scFvs and analysis of the effects of scFvs on T cells

This study was approved by the Ethics Committee in the Pasteur Institute of Iran. After receiving informed consent, fresh heparinized peripheral blood samples were collected from five healthy adult volunteer donors. Using Ficoll–Paque (Sigma) and density gradient centrifugation, peripheral blood mononuclear cells (PBMCs) were isolated from whole blood. Negative selection (depletion of unwanted cells) was used to purify human CD3^+^ T cells from PBMC using magnetic affinity cell sorting (MACS) and the Naive Pan T-Cell Isolation Kit (Miltenyi Biotech). The PBMCs were washed twice with MACS buffer reagent (Miltenyi Biotech), centrifuged at 300×g for 10 min at 20 °C and the supernatant was completely removed. The cell pellet was resuspended in 40 μl of isolation buffer per 10^7^ total cells. Then, to deplete all the non-target cells, a cocktail of biotin-conjugated mAbs against non-T-cell markers was used for their labelling. After incubation, 30 ml buffer and 20 ml Naive Pan T-Cell Micro Bead Cocktail were added (for 10^7^ total cells). Following an additional incubation on ice for 10 min, all the magnetically-tagged unwanted cells were removed by retaining them on the MACS column of a MACS separator. All non-labeled CD3^+^ T cells were washed out from the column and collected in the tube (Fig. 1a) [29, 30].

### T-cell purity assessment using flow cytometry

Isolated T-cell purity was analyzed using flow cytometry with anti-human CD3 monoclonal antibody with fluorescein isothiocyanate (FITC; eBioscience). Briefly, T cells were washed with washing buffer (PBS 0.15 M, 0.5% BSA, 0.1% NaN_3_), resuspended in buffer, and then labeled with anti-CD3 FITC mAb for 40 min at 4 °C in a dark place. The cells were then washed again with the washing buffer. The percentage of CD3^+^ cells was determined using a Partec PASIII flow cytometer (Partec). A minimum of 100,000 events was acquired, and the data analysis was performed using FlowJo software (v.7.2.5; Tree Star).

### T-cell stimulation methods

Comparison of in vitro T-cell stimulation methods was initially performed to obtain optimized stimulation conditions. For this, 10^6^ T-cells in RPMI-1640 containing 15% heat-inactivated fetal bovine serum (FBS; Gibco) were seeded into 24-well plates. Then, T cells were treated for 24 h at 37 °C in 5% CO_2_ with different concentrations of IL-2 (soluble form, R&D Systems), anti-CD3 (soluble form, Mabtech) or PHA (5 μg/ml) as a positive control (Table 1). Cell count and viability were determined using Trypan blue staining [31, 32].

**Table 1:** Summary of the T cell stimulation methods used

NO	IL-2(IU/ml)	Anti-CD3 (ng/ml)
1	100 IU/ml	–
2	200 IU/ml	–
3	300 IU/ml	–
4	–	100 ng/ml
5	–	200 ng/ml
6	–	300 ng/ml
7	100 IU/ml	100 ng/ml
8	200 IU/ml	100 ng/ml
9	300 IU/ml	100 ng/ml
10	100 IU/ml	200 ng/ml
11	200 IU/ml	200 ng/ml
12	300 IU/ml	200 ng/ml
13	100 IU/ml	300 ng/ml
14	200 IU/ml	300 ng/ml
15	300 IU/ml	300 ng/ml

### T-lymphocyte treatment with anti-4-1BB scFvs

To evaluate the impact of anti-4-1BB scFvs on T-cell activation, proliferation and cytokine production, primary T cells were treated with the antibody fragments. Anti-4-1BB scFvs, including PI.12, PI.42, PII.16 and PII.29, were selected and produced as previously described (Fig. 1b). Freshly purified T cells were maintained in RPMI 1640 medium containing 20% FBS, 100 μg/ml streptomycin, and 100 U/ml penicillin. After that, cells were grown for 36 h in the presence of IL-2 and anti-CD3 antibody at 37 °C in a humidified CO_2_ incubator. For cell treatment, 500 × 10^3^ purified T-cells were seeded into 24- and 48-well plates and treated with 10 μg/ml different scFvs for 72 h (Fig. 1c) [28].

### Flow cytometry assessment of T-cell activation marker

To investigate the effect of PI.12, PI.42, PII.16 and PII.29 scFvs on T-cell activation, CD69 upregulation was measured via flow cytometry. In brief, after a 24-h treatment with anti-4-1BB and an unrelated scFv (anti-c-Met scFv named ES1) [33], one million cells were washed with washing buffer, labeled with PE-conjugated anti-CD69 antibody mAb (eBioscience), and analyzed using flow cytometry. Untreated T cells were used as a negative control. A minimum of 100,000 events were acquired. All data analysis was performed using the FlowJo software (v.7.2.5; Tree Star) [28].

### Proliferation assay

To assess the impact of anti-4-1BB scFvs on the proliferation of T cells, cell division analysis was performed by examining 5- and 6-carboxyfluorescein diacetate succinimidyl ester (CFSE) (eBioscience) dilution in labeled cell populations. Briefly, T cells were washed and stained with CFSE reagent at a concentration of 5 μM for 10 min at 37 °C. Then the cells were washed, recounted and treated with scFvs for 72 h at 37 °C. After that, flow cytometry was used to detect CFSE-labeled cells [34].

### Real-time PCR

The mRNA levels of IL-2, IL-4 and IFN-γ were examined using real-time PCR. Briefly, total RNA extraction was performed from T cells treated with scFvs with an RNeasy Mini Kit (Qiagen). After that, transcription into cDNA was performed using 1 mg total RNA and a cDNA synthesis kit (Roche). The expression levels of IL-2, IL-4 and IFN-γ mRNA were quantified with a Two-Step Quanti Test SYBR Green RT-PCR kit (Takara), using a Corbett Rotor-Gene 6000 thermal cycler (Corbett Life Science). Primers for IL-2, IL-4 and IFN-γ were designed using Primer-Blast software (NCBI). Hypoxanthine-guanine phosphoribosyl transferase (HPRT) was used as the housekeeping gene (Table 2). Differences in cytokine mRNA expression levels were determined using the 2 − ^ΔΔCt^ method.

**Table 2:** Primer sequences for real-time PCR

Gene	Primer sequence	
	Forward	Reverse
IL-2	ATGTACACCATGCAACTCCTGTCT	GTCAGTGTTGAGATGATGCTTTGA
IL-4	GCCTCACAGAGCAGAAGAACAC	GTTGGCTTCCTTCACAGGACAG
IFN-γ	GGGTTCTCTTGGCTGTTACT	GAGTTCCATTATCCGCTACATCT
HPRT	AATTATGGACAGGACTGAACGTCTTGCT	TCCAGCAGGTCAGCAAAGAATTTATAGC

### Cytokine analysis via ELISA

The effect of anti-4-1BB scFvs on IL-2, IL-4 and IFN-γ secretion was evaluated in treated T-cells using ELISA. For this, T cells were seeded at 10^5^ cells/well in 96-well cell culture microplates. After 48–72 h incubation with anti-4-1BB scFvs, cytokine levels were measured in cell culture supernatants using ELISA with a cytokine detection kit for IL-2 and IFN-γ from eBioscience and for IL-4 from R&D Systems according to the manufacturer’s instructions. To create standard curves and regression analysis of mean absorbance, standard cytokine solutions were run in parallel. The optical density at 450 nm was measured for each well using a microtiter plate reader (BP-800; Bioship) and the cytokine concentration was obtained from standard curves of recombinant human IL-2, IL-4 and IFN-γ [35].

### Statistical analysis

The statistical difference between two groups was compared with Student’s t-test. Intergroup comparisons were made using the Kruskal–Wallis non-parametric ANOVA test. All data were analyzed using Graph Pad Prism version 6.01 software (Graph Pad). Results are reported as the means ± standard deviation (SD). *p* < 0.05 was considered to be statistically significant.

## Results

### T-cell isolation and stimulation optimization

**Fig. 2:**
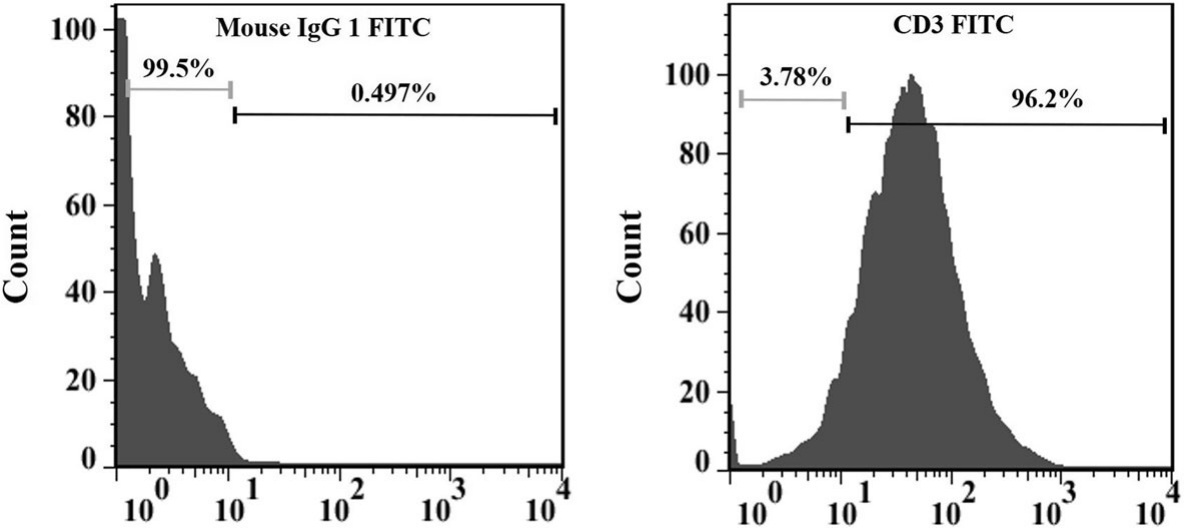
The purity of CD3^+^primary T cells isolated from PBMCs using MACS. The isolated cells were stained with anti-CD3 conjugated to FITC and isotype control antibody and analyzed via flow cytometry. The histograms show the isotype-controlled cells (left) and anti-CD3 stained T cells (right) of the enriched fraction. Results are representative of three independent experiments

Human primary T cells were isolated from PBMC, and their purity was determined using flow cytometry analysis to be about 90% (Fig. 2). Various IL-2 and anti-CD3 antibody concentrations were used to stimulate T cells. It was found that stimulation of T cells with 300 IU/ml IL-2 in combination with 200 ng/ml anti-CD3 antibody resulted in the best cell viability (95%) and proliferation. T-cell viability was about 60% in the absence of stimulatory molecules.

### Anti-4-1BB scFvs induces CD69 expression on T cells

**Fig. 3:**
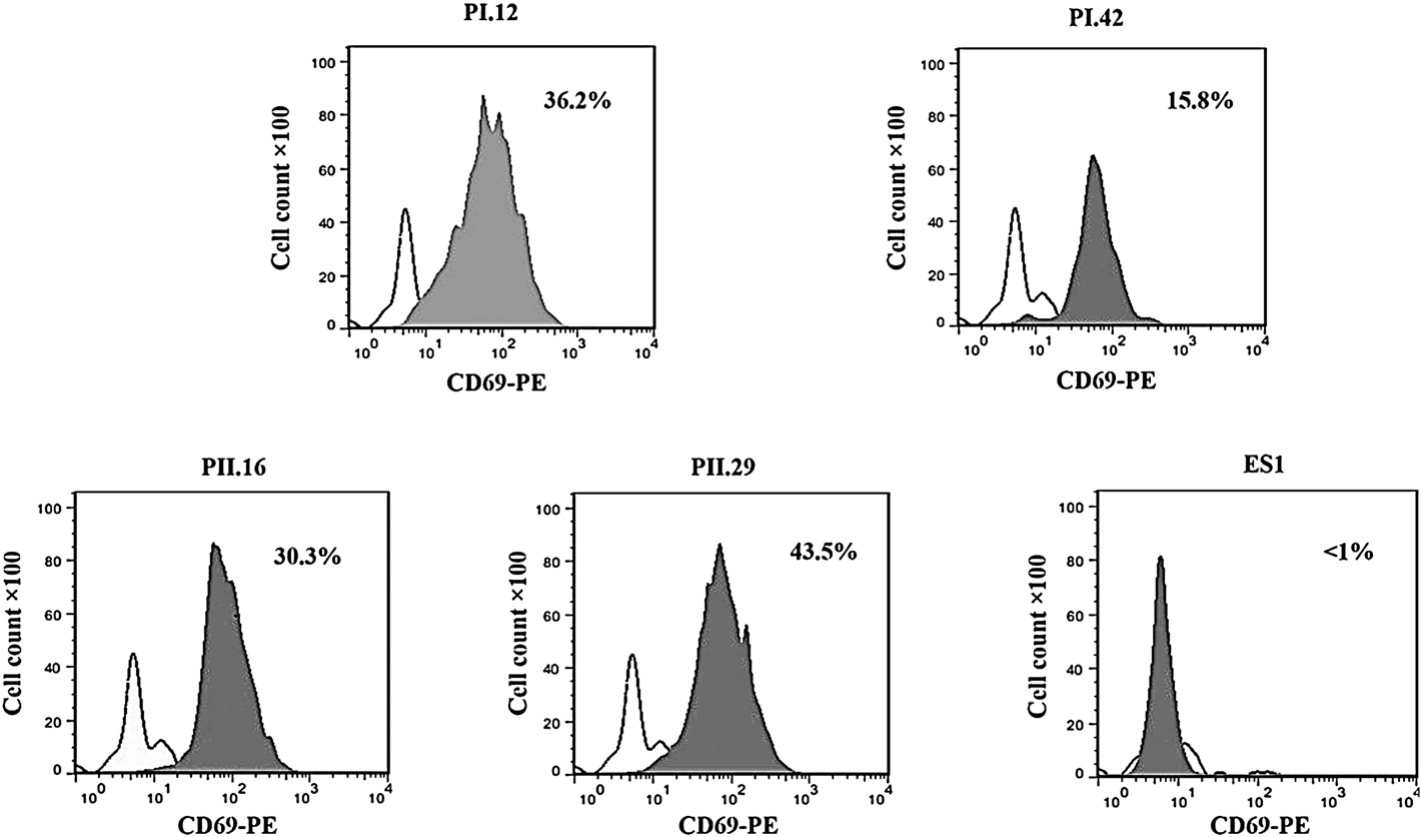
The effects anti-4-1BB scFvs on the expression of the T-cell activation marker CD69. T cells were cultured at 10^6^ cells/well in a 24-well plate, activated with anti-4-1BB scFvs and analyzed for CD69 expression using anti-CD69 PE antibody. The expression level of CD69 increased on T cells activated with PI.12, PI.42, PII.16 and PII.29 scFvs, but not with ES1 control scFv. These results are representative of at least three independent experiments. Dotted line histograms indicate untreated control T cells and gray filled histograms indicate T cells treated with anti-4-1BB and ES1 scFvs

The activation state of T cells was evaluated using flow cytometry analysis of CD69 expression. The expression of CD69 significantly increased on T cells treated with anti-4-1BB compared to that on untreated cells and cells treated with control scFv (ES1; *p* < 0.05; Fig. 3). The average expression increased from 1.5 ± 0.2% on non-treated to 36.2 ± 2.0, 15.8 ± 1.1, 30.3 ± 2.1 and 43.5 ± 2.0% for cells treated with PI.12, PI.42, PII.16 and PII.29 scFvs, respectively.

### Anti 4-1BB scFv enhances T-cell division and proliferation

**Fig. 4:**
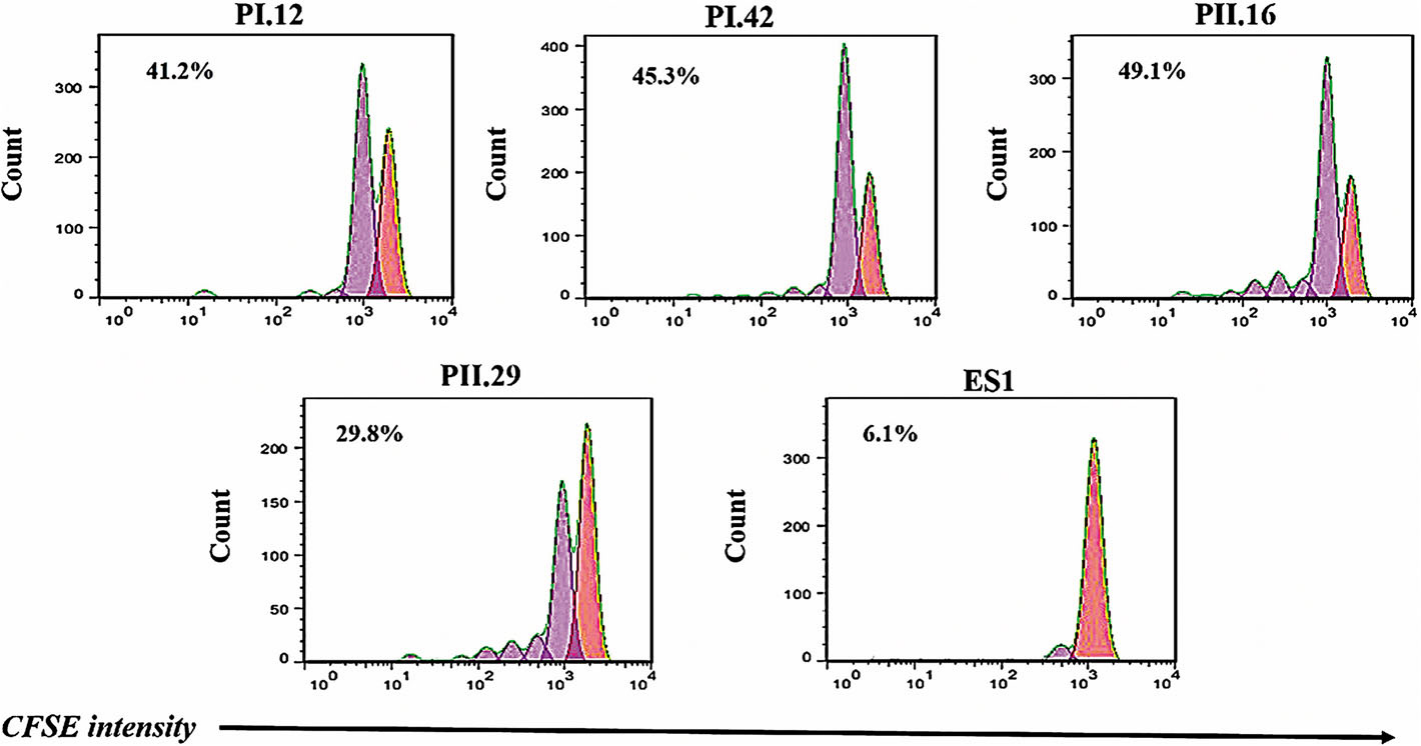
Induction of cell division by anti-4-1BB scFvs. To measure cell division, purified T cells were labeled with CFSE and treated as described in the Materials and Methods section. After 72 h of treatment, the T cells were stained with CD3 and analyzed for CFSE levels via flow cytometry. Representative data show the % division and are representative of three independent experiments. The decrease in the mean fluorescence intensity of the CFSE-labeled cells was observed in cells treated with anti-4-1BB scFvs, which indicates that cell division had taken place. No division was observed in cells treated with ES1 and control (*p* < 0.05)

CFSE labeling was used to determine whether anti-4–1BB scFvs increase the ability of T cells to divide. T cells stimulated with IL-2 and anti-CD3 were stained with CFSE, incubated with or without scFvs for two days, and analyzed for the number of divisions undergone. All scFvs examined showed clear evidence of cell division compared to the unrelated scFv (Fig. 4). Treatment with PI.12, PI42, PII.16 and PII.29 anti-4-1BB scFvs increased the cell division (*p* < 0.05) to 41.2, 49.9, 45.3 and 29.8%, respectively. These results show that anti-4-1BB scFvs could provide a costimulatory signal to induce cell division in T cells.

### The mRNA expression of IL-2and IFN-γ increases in treated T cells

**Fig. 5:**
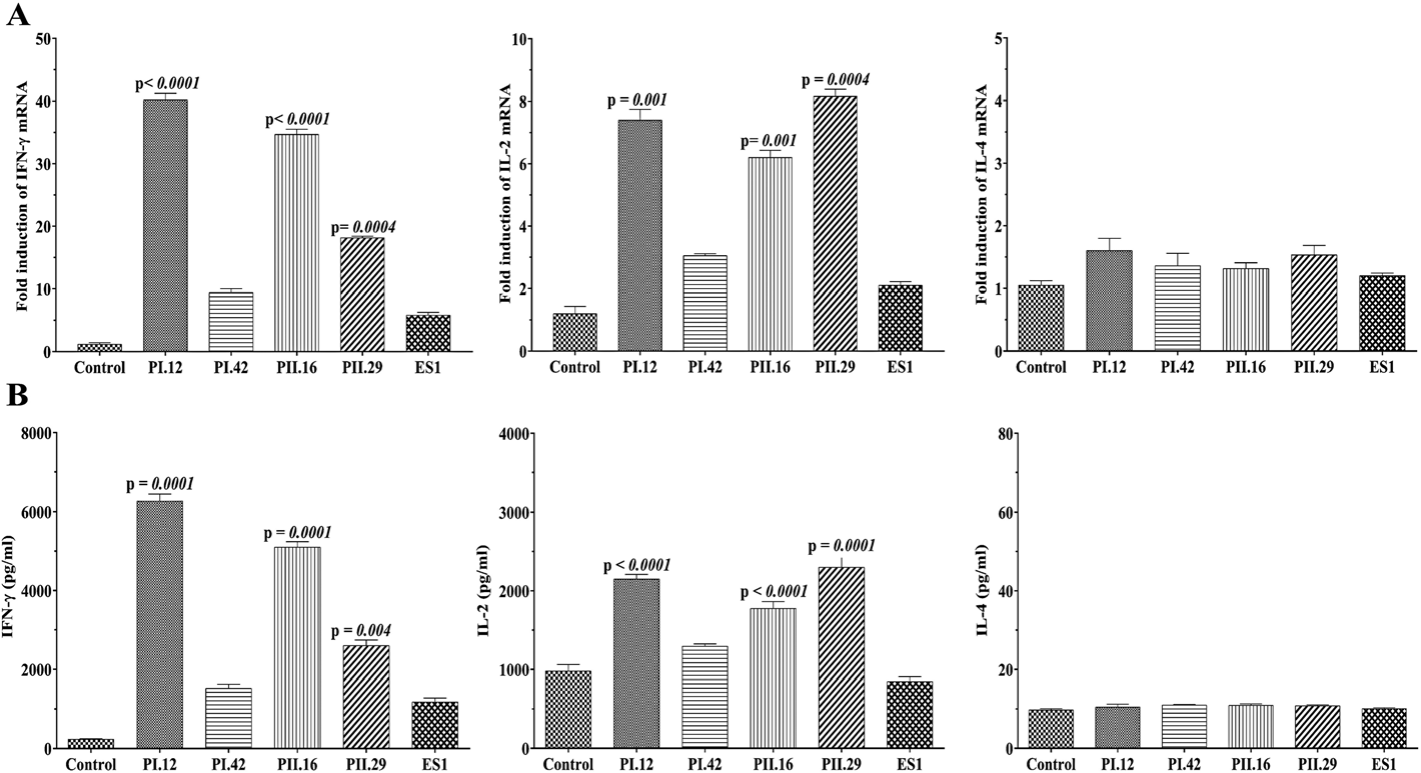
Cytokine mRNA expression and enhanced IFN-γ and IL-2 production in T cells treated with anti-4-1BB scFvs. **a** Following 72 h of treatment with anti-4-1BB scFvs, cells were collected and analyzed for IFN-γ, IL-2 and IL-4 mRNA expression levels using real-time PCR, with the amplification normalized against HPRT. Results from quantitative real-time PCR demonstrate that mRNA for IFN-γ and IL-2 was significantly increased in T cells treated with PI.12, PII.16 and PII.29 scFvs.PI.42 and that ES1 scFvs could not induce IFN-γ and IL-2 production in T cells. No IL-4 mRNA expression was observed in the treated cells. NS: no significant difference. *p < 0.05. Data represent the mean of three experiments ± SD. **b** Cytokine secretion by T-cells treated with anti-4-1BB scFvs was determined using ELISA. IFN-γ and IL-2 increased significantly following treatment of T cells with PI.12, PII.16 and PII.29 scFvs (*p* < 0.05), while the increase in IL-4 production was not significant. The results shown are means ± SD amounts of cytokine secreted by treated T cells (in pg/ml). Each experiment was repeated a minimum of three times

The effect of anti-4-1BB scFvs on the expressions of IL-2, IL-4 and IFN-γ mRNA was examined via real-time PCR using specific primers. A significant increase in the mRNA expression levels of IFN-γ and IL-2 was observed after 48 h treatment with scFvs (Fig. 5). PI.12, PII.16 and PII.29 anti-4-1BB scFvs respectively increased the IFN-γ mRNA expression from 1.00 ± 0.15 in the control cells to 42.7 ± 2.1 (*p* < 0.0001), 34.4 ± 1.48 (*p* < 0.0001) and 16.02 ± 1.15 (*p* = 0.0004) in the treated cells. Moreover, PI.42 (10.4 ± 1.1) and control scFv (7.9 ± 1.08) could not significantly alter the expression of IFN-γ mRNA in the treated cells (Fig. 5a).

Furthermore, IL-2 mRNA expression increased from 1.2 ± 0.2 to 7.4 ± 0.3 (*p* = 0.001), 6.2 ± 0.2 (*p* = 0.001) and 8.15 ± 0.25 (*p* = 0.0004) in T cells treated with PI.12, PII.16 and PII.29 scFvs, respectively, and no significant difference of IL-2 expression was observed in PI.42-treated (3.1 ± 0.1) and control scFv (2.2 ± 0.15) cells (Fig. 5a). Anti-4-1BB scFvs had no significant effect on the mRNA level of IL-4 in the T cells (Fig. 5a).

### Anti-4-1BB scFvs induced IL-2 and IFN-γ production in T cells

The levels of IL-2, IL-4 and IFN-γ secreted by treated T cells were determined using ELISA. IFN-γ production increased from 211.8 ± 14.12 pg/ml in the control group to 6210 ± 112 (*p* = 0.0001), 5050 (± 103 (*p* = 0.0001) and 2610 ± 101 (*p* = 0.004) pg/ml in the PI.12-, PII.16- and PII.29-treated cells, respectively. PI.42 increased IFN-γ production (1425 ± 75) but the difference was not significant. Also, ES1 was not able to induce IFN-γ production in treated cells (1180 ± 70; Fig. 5b). In addition, the treatment of T cells with anti-4-1BB scFvs led to a significant increase in the secretion of IL-2 by T cells compared with the untreated control group (Fig. 5b). The results showed that anti-4-1BB scFvs had no effects on the production of IL-4 by T cells (Fig. 5b).

## Discussion

Cancer treatment strategies include chemotherapy, radiotherapy and immunotherapy, which can be applied individually or in combination. ACT has emerged as a realistic technique for cancer treatment [36, 37].

However, the success of adoptive T-cell therapy has been limited to certain tumor types, in part due to tumor-induced T-cell inactivation [18, 38, 39]. Several strategies have been exploited to improve the proliferative potential and therapeutic efficacy of T lymphocytes for adoptive cell therapy. Agents that enhance the anti-tumor functions of immune cells are a promising novel group of immunotherapeutics. mAbs acting as checkpoint inhibitors or co-receptor agonists have been used to enhance the survival and activation of the T cells [18, 23].

In this study, we used the agonistic anti-4-1BB scFvs PI.12, PI.42, PII.16 and PII. 29, which were produced and selected as previously described. T cells were isolated from the human peripheral blood using the MACS technique and stimulated with IL-2, anti-CD3 mAb and anti-4-1BB scFvs. The effect of anti-4-1BB scFvs treatment was investigated via flow cytometry analysis of CD69 expression, determination of IL-2, IL-4 and IFN-γ production, and T-cell proliferation assay.

It was shown that T-cell treatment with anti-4-1BB scFvs enhanced the expression of T cell activation marker (CD69) on the surface of T cells (Fig. 3) and induced IL-2 and IFN-γ mRNA expression and protein production, but did not increase IL-4 production (Fig. 5a and b). It also obviously increased the proliferation capability of T cells (Fig. 4). The results of IL-2 production and CD69 expression in T cells concur with the results our previous study in which IL-2 and CD69 expression were evaluated in treated CCRF-CEM cells.

In a similar study by Hernandez-Chacon et al., the effect of anti-4-1BB mAb on the proliferation of TILs was investigated [23]. 4-1BB signaling mediated by anti-4-1BB mAb could facilitate the continued proliferation of TIL after TCR-CD3 stimulation and prevent AICD, probably by regulating BCL-2 family proteins, thereby serving as an alternative costimulatory pathway in TIL used for ACT. Wen et al. [34] reported that 4-1BBL could stimulate both CD4 and CD8 T cells and induce T-cell expansion. Cooper et al. showed that 4-1BB enhances the proliferation and survival of cytotoxic T cells in vivo [40]. Shuford et al. exmined in vivo effects of anti-4-1BB mAbs on antigen-induced T-cell activation and showed that 4-1BB triggering preferentially enhances CD8 T cell expansion and contributes to the induction of cytotoxic T-cell responses [41]. Daniel-Meshulam et al. found that T-cell stimulation by 4-1BB can increase IFN- γ production and enhance anti-tumor activity [31]. Miller et al. showed that the 4-1BB-specific monoclonal antibody triggers tumor immune responses through IFN-γ induction.

Previous studies also demonstrated that targeting 4-1BB could improve T-cell responses and mediate anti-tumor activity. The results of Hernandez-Chacon et al. indicate that the provision of 4-1BB costimulation through monoclonal antibodies could improve the survival of TIL during melanoma ACT and boost anti-tumor effector functions [23]. In another study, it was demonstrated that the addition of an agonistic anti-4-1BB antibody to the Rapid Expansion Protocol (REP) could enhance the frequency and total yield of tumor-reactive CTLs and increase their anti-tumor activity and survival capability when re-cultured with or without cytokines [42].

It is well documented that the cross-linking of 4-1BB via ligand-induced trimerization is essential for inducing strong downstream signaling [43]. Therefore, the previous studies used mAbs or [44] multivalent scFvs to [45] trigger the 4-1BB signaling pathway. By contrast, the monovalent 4-1BB specific scFvs used in this study could induce the 4-1BB signaling pathway alone. Moreover, it has been reported that some monovalent scFvs against other TNFR superfamily members could induce receptor cross-linking and initiate cell signaling [46–48]. This might be because of the different binding sites of the scFvs from those of 4-1BBL on 4-1BB that lead to induction of different conformations in the 4-1BB intracellular domain and subsequently different signaling outcomes. Like other TNFR families, each 4-1BB is composed of four cysteine-rich domains (CRDs), CRD1 to CRD4. It exists on the T-cell surface in both monomeric and dimeric forms. It has been reported that 4-1BBL and utomilumab, an agonistic anti-4-1BB mAb, have completely different binding areas on 4-1BB. The binding region of 4-1BBL is located on CRD2 and CRD3, while utomilumab binds to dimeric 4-1BB via CRD3 and CRD4 with partial contacts to CRD2 [49, 50]. Moreover, cross-linking of 4-1BB by utomilumab is relegated by the ratio of mono- to di-4-1BBs which is different from trimeric 4-1BBL [51].

These scFvs have been isolated from a fully human phage display library, so they will not elicit immune responses in the patients. In comparison to the previously studied full-length anti-4-1BB antibodies, these novel single-chain antibodies have many advantages, including smaller size, lower cost and ease of production, which make them suitable for targeting 4-1BB in ACT and for use in a variety of studies.

## Conclusions

The novel human anti-4-1BB scFvs improved various T-cell functional activities, including enhanced cell division, and increased IL-2 and IFN-γ production. Since the scFvs could increase Type I (IL-2 and IFN-γ), but not Type II cytokines (IL-4), it can be concluded that they may induce T-cell-mediated Type I immune responses.

In recent years, substantial progress has been achieved in elucidating the therapeutic potential of anti-4-1BB antibodies alone or in conjunction with other FDA-approved immunomodulatory substances. Current clinical trials on 4-1BB agonists proved the importance of 4-1BB as an emerging therapeutic target. Although our results provide evidence for the immunomodulation advantages of anti-41BB scFvs, further studies are needed to evaluate their in vivo T-cell activation and their anti-tumor effects.

## Data Availability

The datasets used and/or analyzed during this study are available from the corresponding author on reasonable request.

## Abbreviations

ACT: Adoptive cell therapy
AICD: Activation-induced cell death
TNFR: Tumor necrosis factor receptor
scFv: Single-chain fragment variable
PBMCs: Peripheral blood mononuclear cells
MACS: Magnetic affinity cell sorting
CFSE: 5-and 6-carboxy fluoresceindiacetatesuccinimidy ester
APCs: Antigen-presenting cells
HPRT: Hypoxanthine-guanine phosphoribosyl transferase
TCR: T-cell receptor
CAR: Chimeric antigen receptor
FBS: Fetal bovine serum
Ig: Immunoglobulin
IL-2: Interleukin-2
LPS: Lipopolysaccharides
ELISA: Enzyme-linked immunosorbent assay
mAb: Monoclonal antibody
PBS: Phosphate-buffered saline
BSA: Bovine serum albumin
RPMI-1640: Roswell Park Memorial Institute medium
PHA: Phyto hemagglutinin
IFN-γ: Interferon gamma
NF-κB: Nuclear factor kappa-light-chain-enhancer of activated B cells
BCL-2: B-cell lymphoma 2
TNF-α: Tumor necrosis factor-α
